# Supplementary material for: Stakeholders’ perspectives on capturing societal cost savings from a quality improvement initiative: A qualitative study
Source: PLoS One. 2024 Sep 23;19(9):e0310799. doi: 10.1371/journal.pone.0310799 (PMC11419338; doi:10.1371/journal.pone.0310799)
Supplement: S2 File — (DOCX) [file pone.0310799.s002.docx]

**Topic guide**

| Introduction | - What is your position? - What are your thoughts on the prehabilitation program? - Did you observe any changes or effects of the prehabilitation program? |
| --- | --- |
| Excess capacity | The aim of prehabilitation is to reduce complication and consequently the length of stay. If this goal is achieved, it would lead to savings in hospital capacity.   - What is the impact of reducing the length of stay on your workload?      - What do you expect to happen with the newly available capacity? Can you provide an example where this happened? - Under what conditions can a bed remain unoccupied and not be refilled?   - What can facilitate this?   - What may preventing this? - Are there incentives to provide more care?   - At the hospital level   - At the department level   - At the level of the healthcare professionals? |
| Downsizing | - Would you consider downsizing of in case substantial capacity savings?   - What are reasons to do so?   - What are reasons not to do so?   - How would you feel if it happens? - Has labor shortages influenced the department’s capacity?   - What do you expect for the near future? |
| Reducing department expenses | - Could your department reduce expenses if prehabilitation is effective?   - What can facilitate this?   - What may preventing this?   - Can you provide an example where this happened? - When would you be able to scale down staff?   - Why would you do it? Why wouldn’t you do it?   - How would you feel if it happens? - What is the impact on the department’s revenue when patients are discharged earlier? - What is the impact on the department’s revenue when more new patients are admitted? - How do the department’s revenues relate tot he hospital’s revenues? |
| Reducing hospital expenses | - How can providing less care to a patients result in cost savings for the hospital? - Could the hospital reduce expenses if prehabilitation is effective?   - What can facilitate this?   - What may preventing this?   - Can you provide an example where this happened? |
| Reducing societal expenses | - How can hospital cost savings be translated into savings for society? - Under what circumstances would the hospital be willing to pass on cost-savings to health insurers? - How can hospital cost savings be transfered to insurers   - What can facilitate this?   - What may preventing this? - Are initiatives like prehabilitation considered in agreement negotiations? |
| Conclusion | - Is there anything you like to add? |
